# The possible involvement of intestine-derived IgA_1_: a case of IgA nephropathy associated with Crohn’s disease

**DOI:** 10.1186/s12882-016-0344-1

**Published:** 2016-09-05

**Authors:** Tomohiro Terasaka, Haruhito A. Uchida, Ryoko Umebayashi, Keiko Tsukamoto, Keiko Tanaka, Masashi Kitagawa, Hitoshi Sugiyama, Hiroaki Tanioka, Jun Wada

**Affiliations:** 1 Department of Nephrology, Rheumatology, Endocrinology and Metabolism, Okayama University Graduate School of Medicine, Dentistry and Pharmaceutical Sciences, 2-5-1 Shikata-cho, Kita-ku, Okayama, 700-8558 Japan; 2 Department of Chronic Kidney Disease and Cardiovascular Disease, Okayama University Graduate School of Medicine, Dentistry and Pharmaceutical Sciences, 2-5-1 Shikata-cho, Kita-ku, Okayama, 700-8558 Japan; 3 Department of Human Resource Development of Dialysis Therapy for Kidney Disease, Okayama University Graduate School of Medicine, Dentistry and Pharmaceutical Sciences, 2-5-1 Shikata-cho, Kita-ku, Okayama, 700-8558 Japan; 4 Department of Medicine Oncology, Okayama Rosai Hospital, 1-10-25 Chikkomidori-machi, Minami-ku, Okayama 702-8055 Japan

**Keywords:** Crohn’s disease, IgA nephropathy, IgA_1_, IgA_2_, Inflammatory bowel disease

## Abstract

**Background:**

A link between IgA nephropathy and Crohn’s disease has recently been reported. Other researchers hypothesize that intestine-derived IgA complexes deposit in glomerular mesangial cells, eliciting IgA nephropathy. Intestinal mucosal plasma cells mainly secrete IgA_2_. Nevertheless, IgA_1_ deposition is strongly implicated as being the primary cause of IgA nephropathy.

**Case presentation:**

A 46-year-old Japanese man developed IgA nephropathy 29 years ago, following tonsillectomy. As a result, a normal urinalysis was obtained. The patient previously suffered Crohn’s disease followed by urinary occult blood and proteinuria six years ago. Exacerbation of IgA nephropathy was highly suspected. Therefore a renal biopsy was performed. A diagnosis of exacerbation of IgA nephropathy with mesangial cell proliferation and fibrotic cellular crescent was based upon the pathological findings. The patient exhibited a positive clinical course and eventually achieved a remission with immunosuppressive therapy including prednisolone treatment. Immunostaining for the detection of IgA subtypes was performed on both of his kidney and excised ileum. The results revealed IgA_1_ and IgA_2_ deposition by submucosal cells in intestine. Furthermore, IgA_1_ deposition of mesangial areas in the patient’s kidney, indicated an association of IgA_1_ with the exacerbation of IgA nephropathy.

**Conclusion:**

This case represents the possibility that the intestine-derived IgA_1_ can be the origin of galactose-deficient IgA which is known to cause IgA nephropathy exacerbation.

## Background

IgA_1_ is one of the subtypes of IgA produced in bone marrow, tonsils and respiratory tracts. Also, it can be found predominantly in the glomeruli of patients with IgA nephropathy. This observation supports the idea of using tonsillectomy as an effective therapeutic intervention for IgA nephropathy. Alternatively, intestinal mucosal plasma cells mainly secrete IgA_2_ (approximately 60 % in mucosal cells), another subtype of IgA, rather than IgA_1_ [1].

Recent reports have indicated a strong link between IgA nephropathy and Crohn’s disease. Several hypotheses exist, indicating this, however the underlying mechanism is still unknown. One can speculate that intestine-derived IgA is possibly deposited in glomerular mesangial cells, eliciting IgA nephropathy [2–4]. Here, we present a case report of a patient suffering from exacerbated IgA nephropathy associated with Crohn’s disease, who had tonsillectomy 29 years ago. We performed immunohistochemical analysis in renal biopsy and excised ileum specimen to determine whether it was IgA_1_ or IgA_2_ that was specifically associated with the exacerbation of IgA nephropathy. The results indicated that IgA_1_, not IgA_2_, is the causative source of IgA nephropathy exacerbation. This indicates a link between inflammatory intestine-derived IgA_1_ and IgA nephropathy.

## Case presentation

**Fig. 1:**
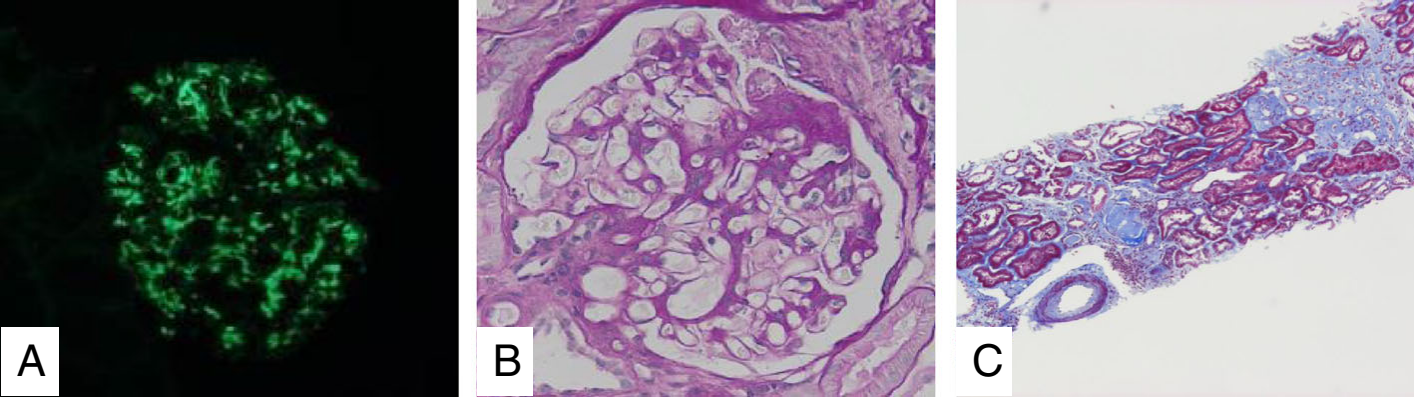
Histological findings of the renal tissues. **a** IgA, (**b**) Mesangial matrix expansion and fibrocellular crescent formations were observed in glomeruli with periodic acid-Schiff (PAS) staining (400×). **c** Slight changes of interstitial fibrosis and arteriole sclerosis with Masson staining (40×)

A 46-year-old Japanese man suffered from IgA nephropathy, prior to be given tonsillectomy 29 years ago. Urinalysis revealed neither hematuria nor proteinuria after surgical removal of his tonsils. The patient experienced a sudden onset of Crohn’s disease 6 years ago. As a result, he was administered several medications to alleviate the symptoms of Crohn’s disease; initially, 5-aminosalicylic acid (ASA). Due to the unstable state of Crohn’s disease, an ileum resection was performed. Additionally, prednisolone, azathioprine (AZA) and infliximab were eventually incorporated in his treatment. Despite therapeutic drug intervention, his abdominal symptoms including diarrhea persisted. Consequently, he was then referred to our hospital because of proteinuria and urinary occult blood. An otolaryngologic check-up revealed no sinusitis, and residual tonsil and gross physical examination provided no remarkable findings. Abdominal CT displayed bilateral slightly atrophic kidneys. A laboratory workup showed a mild renal dysfunction (creatinine; 1.37 mg/dl, creatinine clearance; 69.1 ml/min), without any other abnormal findings of blood (IgG, 1492.3 mg/dl; IgA, 223.2 mg/dl; IgM, 33.8 mg/dl; C3, 101.4 mg/dl; C4, 22.7 mg/dl; CRP, 0.09 mg/dl, anti-streptolysin O, 109 IU/ml; normal range: 0–239; anti-streptokinase, ×1280; normal: < ×2560). Serologic tests for hepatitis B and C and HIV were all negative. Urinalysis showed proteinuria (2+) and occult blood (2+), with 5–9 red blood cells and < 1 white blood cells per high-power field in sediment. Twenty four-hour urine collection revealed a protein level of 1.44 g/day. To detect occult blood (1+ to 2+) and continuous proteinuria (2+ to 3+), a renal biopsy was performed to examine whether IgA nephropathy relapsed or other glomerular diseases occurred. Immunofluorescent examination of mesangial cells exhibited intense (3+) mesangial granular staining of IgA (Fig. 1a). Moderate (2+) granular staining of IgM and C3 in the mesangial area was observed. Examination by light microscopy revealed expansion of mesangial cells and fibrotic cellular crescents (Fig. 1b). These findings are consistent with IgA nephropathy. Given these observations, prednisolone of 40 mg/day was started, with 10–20 % reduction monthly and with every subsequent visit. After 1-year of treatment (5 mg/day dosage of prednisolone is continued), his urine protein levels decreased to 0.1 to 0.3 g/gCr, with no occult blood was detected.

**Fig. 2:**
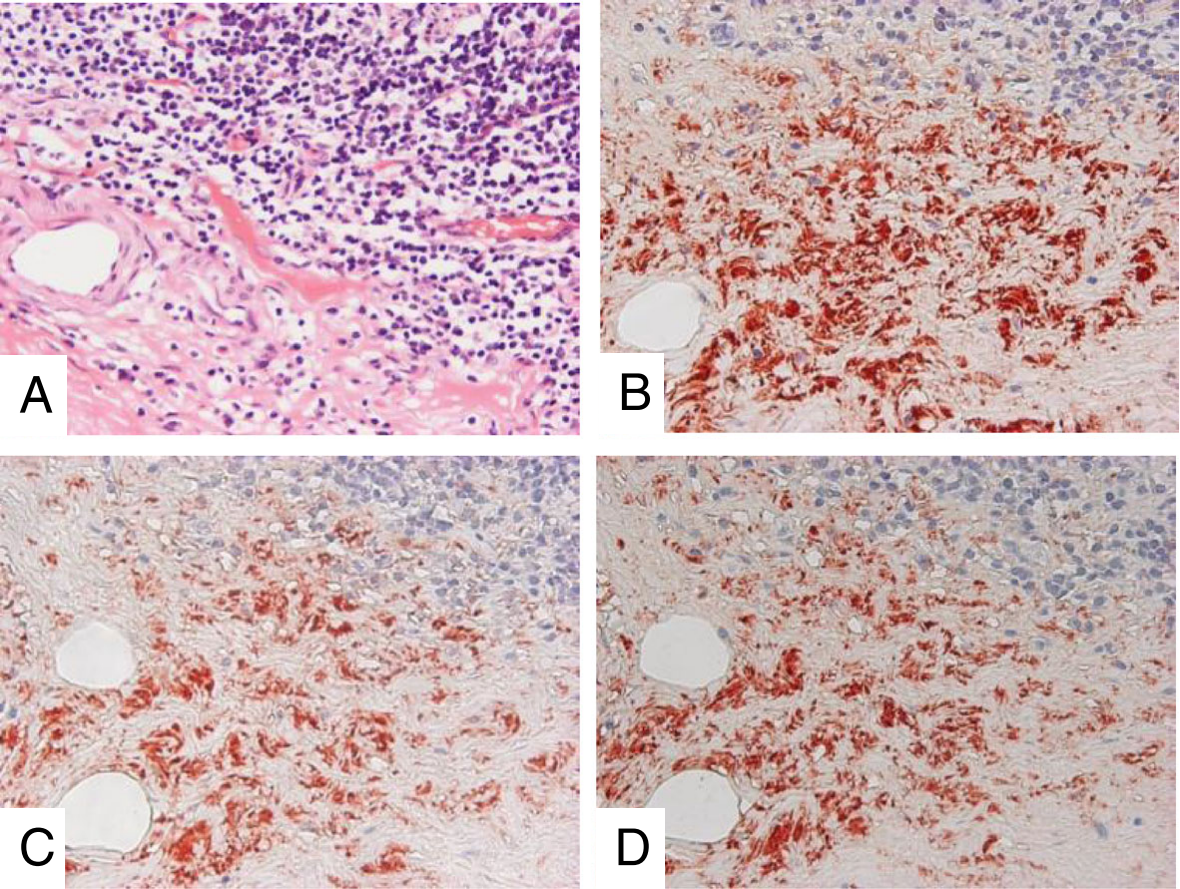
Immunohistological findings of the intestine tissues. Exacerbated intestine mucosal infiltration of lymphocytes and plasma cells with Hematoxylin-Eosin staining (**a**). Immunostaining of total IgA (**b**), IgA_1_ (**c**), IgA_2_ (**d**) in submucosal layer of intestine

**Fig. 3:**
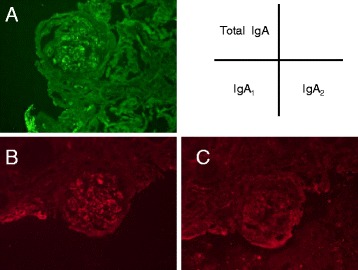
Immunofluorescent histological findings of the glomeruli. Immunostaining of total IgA (**a**), IgA_1_ (**b**), IgA_2_ (**c**) in kidney glomeruli

Due to his clinical course, we strongly suspected the association between the occurrence of intestinal inflammatory disease and the exacerbation of IgA nephropathy. Based on the predominance of IgA_2_ subtype found in the intestinal immune system, the patient’s excised ileum and biopsied kidney species were examined to determine whether IgA_2_ subtype might be mainly involved in the exacerbation of IgA nephropathy. Immunohistochemical staining demonstrated that lymphocytes and plasma cells had infiltrated on the inflammatory ileum mucosa. In particular, at the layer of submucosa, total IgA, IgA_1_ and IgA_2_ positive cells were widely distributed, a number of IgA_1_ positive cells were observed as well as IgA_2_ positive cells (Fig. 2). Alternatively, the patient’s kidney sample demonstrated that IgA_1_ was mainly deposited granular to mesangial areas (Fig. 3). These results raised the possibility that inflammatory ileum derived IgA_1_ in our patient suffering from Crohn’s disease was in fact associated with the exacerbation of IgA nephropathy.

## Conclusions

Renal or urologic complications are common in Crohn’s disease (up to 25 % of diagnosed patients) [5]. However, most of patients affected with Crohn’s disease exhibit urologic complications, such as nephrolithiasis with calcium oxalate or urate stones, enterovesical fistulas and ureteral obstruction [6]. Renal parenchymal dysfunction of tubulointestitial nephritis (TIN) occurs in 1 out of 500 patients who undertake 5-ASA, which is one of the key drugs prescribed for inflammatory bowel disease (IBD) [7]. 5-ASA induced TIN is reported to appear mostly within 12 months of the drug administration, but the prolonged phase of 5-ASA treatment also can cause TIN. Early and definite diagnosis of nephropathy with IBD should be confusing because their cases are generally medicated with 5-ASA for a long time.

IgA nephropathy is the most common type of glomerulonephritis and it is caused by primary or secondary pathogen disease such as seronegative arthritis, cirrhosis, celiac disease, vasculitis and HIV [8]. Although the effect of tonsillectomy on IgA nephropathy is still controversial, several reports suggest its practical efficacy at the present time. The retrospective study showed that tonsillectomy had an effect on renal outcome [9]. The decrease level of proteinuria in steroid pulse therapy with or without tonsillectomy was significant [10]. Reports of IgA nephropathy associated with Crohn’s disease are increasing. A retrospective review of 83 kidney biopsies with IBD report that the most common diagnosis was IgA nephropathy (24 %), followed by interstitial nephritis (19 %) which was suspected of 5-ASA relation, and arterionephrosclerosis (12 %) [11]. In that review, it was not clearly mentioned whether the occurrence of IgA nephropathy in Crohn’s disease was different from that in ulcerative colitis. The mechanism of the complication between IgA nephropathy and Crohn’s disease has been discussed in three points. First, the systemic absorption of IgA at the intestine mucosal inflammation site is associated with IgA nephropathy [2–4]. Second, the possible involvement of a common genetic factor; especially human leukocyte antigen (HLA)-DR1 has been the suspected factor that provides a link between Crohn’s disease and IgA nephropathy [12, 13]. Third, abnormal T helper lymphocyte is a factor that may contribute to the pathogenesis associated with these two diseases [14].

Further experiments are needed to confirm these hypotheses. On the other hand, in Crohn’s disease, intestinal mucosal inflammation is thought to lead to the systemic absorption of antigens and bacteria, and high serum levels of IgA and IgG [2]. Alternatively, increased intestinal mucosal permeability was demonstrated in IgA nephropathy [15]. The association of the etiology of Crohn’s disease after tonsillectomy has been reported [16], yet, it is not clear whether this association is applicable to our case or not. During the term of exacerbation of bowel disease, IgA nephropathy deteriorates or clinically evoked. Treatment of bowel disease with either immunosuppression therapy or bowel resection is associated with a clinical remission of IgA nephropathy [17]. These clinical observations support that a high level of systemic IgA derived from the ileum tract leads to the onset of IgA nephropathy. We hypothesized that intestine-derived IgA_2_ would react to this mechanism, since mucosa of lower gastrointestinal tract secreted mostly IgA_2_ than IgA_1_. Nonetheless, in this case, IgA_1_ deposition, but not IgA_2_ deposition, in kidney glomerular mesangial sites was observed. The galactose-deficient IgA_1_ is recognized as being the likely cause of IgA nephropathy [18, 19]. IgA_1_ producing cells are known to increase in the intestine of IBD [20], which also provides the potential answer to why intestine-derived abnormal sugar-chain IgA_1_ is a causative agent of IgA nephropathy. In conclusion, there appears to be a link between Crohn’s disease, IgA nephropathy and galactose-deficient IgA_1_ secreted by the intestine, and all may be confounding factors that contribute to the onset or exacerbation of IgA nephropathy.

## Abbreviations

ASA: 5-aminosalicylic acid
AZA: Azathioprine
HLA: Human leukocyte antigen
IBD: Inflammatory bowel disease
IgA: Immunoglobulin A
IgG: Immunoglobulin G
IgM: Immunoglobulin M
TIN: Tubulointerstitial nephritis
